# Cannabis-only use in the USA: prevalence, demographics, use patterns, and health indicators

**DOI:** 10.1186/s42238-022-00143-y

**Published:** 2022-07-22

**Authors:** Beatriz H. Carlini, Gillian L. Schauer

**Affiliations:** 1 Psychiatry and Behavioral Sciences Department, Addictions, Drug & Alcohol Institute, University of Washington, Seattle, WA USA; 2 Department of Health Systems and Population Health, School of Public Health, University of Washington, Seattle, WA 98105 USA

**Keywords:** Cannabis, Marijuana, Cannabis and polydrug use, Cannabis-only use

## Abstract

**Background:**

The prevalence of adults who consume cannabis while abstaining from other substances is not known in the USA. This study used nationally representative data to estimate the prevalence and explore the demographic characteristics, cannabis use behaviors, and self-reported health of US adults with past 30-day cannabis-only use, as compared with adults who used cannabis as well as other substances.

**Methods:**

Data came from adults 18 years and older who responded to the 2017 or 2018 National Survey on Drug Use and Health and reported past 30-day cannabis use (*n* = 12,143). Cannabis-only use was defined as past 30-day cannabis use with no past 30-day use of other substances (alcohol, tobacco, other illicit substances, non-prescribed controlled medications). Weighted frequencies and 95% confidence intervals (CI) were computed for all sociodemographic and cannabis-related variables, overall, and across the two categories of cannabis consumers, stratified by age.

**Results:**

The prevalence of past 30-day cannabis-only use among US adults was 0.9% (95% *CI*: 0.8, 1.0) and varied by age (2.0% of 18–25 years old; 0.7% of 26–49 year olds, and 0.6% of those ≥ 50 years). Among adults with any past 30-day cannabis use, 8.4% (95% *CI*: 7.6, 9.2; *n* = 980) reported cannabis-only use. Age was significantly associated with past 30-day cannabis-only use, with adults 18–25 years and 26–49 years having higher odds of cannabis-only use compared with older adults. Past year cannabis dependence was lowest among cannabis-only consumers aged ≥ 50 years (0.2%; 95% *CI*: 0.1, 0.5) and highest among young adult cannabis and other substance consumers (16.7%; 95% *CI*: 15.3, 18.2). Past year prevalence of any mental illness was generally similar across cannabis use groups and by age.

**Conclusions:**

The prevalence of adult cannabis-only use in the US is low — most cannabis consumers report using other substances in the past 30 days as well. While cannabis-only use among older adults is rare, it does not appear to be accompanied by a high prevalence of dependence. These findings should guide future research and policymaking.

## Background

More than 18 US states have now legalized adult use of cannabis (National Conference of States Legislatures 2022), a historical shift in drug policy not experienced since the repeal of alcohol prohibition in the 1932 (National Research Council (US) 1981).

Cannabis industry advocates and individuals who use cannabis medicinally have emphasized cannabis use to achieve well-being and to cease use of other harmful substances (like opioids) (Barcott 2016; Dutcher 2018; Stukin 2019). On the other hand, prevention, treatment, and public health professionals have expressed concerns about widespread acceptance of a substance that can be addictive and that has been associated with a range of health risks, including increased dependence on and potential use of other substances (National Academy of Sciences 2017; Crocker et al. 2021; Hall 2018).

Understanding the prevalence and characteristics of those who use cannabis alone versus those who use cannabis and other substances is important to inform public education and programming that seeks to protect public health and consumer safety, as the risk profile of cannabis-only use may differ from polydrug use. Accordingly, this study used nationally representative data to estimate the prevalence and explore the demographic characteristics, cannabis use behaviors, and self-reported health of US adults with past 30-day cannabis-only use, as compared with adults who used cannabis as well as other substances.

## Methods

### Sample

Data for this study came from 12,143 noninstitutionalized adults age 18 and older who responded to the National Survey on Drug Use and Health (NSDUH) between 2017 and 2018 and reported past 30-day cannabis use. NSDUH is a nationally representative household survey sponsored by the Substance Abuse and Mental Health Services Administration (SAMHSA) and conducted annually with US civilians aged 12 years and older. NSDUH uses stratified, independent, multistage area probability-based sampling within each state and the District of Columbia. Overall weighted response rates (screening response rate × interview response rate) for the 2017 and 2018 NSDUH were 50.4% and 48.8%, respectively. Weighted interview response rates were 67.1% and 66.6%, respectively. Interviews are conducted using computer-assisted personal interviewing (CAPI). Answers to sensitive questions are gathered using audio computer-assisted self-interview (ACASI). More methodological details about NSDUH can be found elsewhere (Center for Behavioral Health Statistics and Quality 2018, 2019).

### Measures

#### Cannabis-only use and cannabis use with other substances

“Cannabis-only” use was defined as reported past 30-day use of cannabis (to include marijuana, hashish, or blunts) and no reported past 30-day use of other substances measured as part of NSDUH (i.e., alcohol, illicit drugs (including use cocaine, crack, heroin, hallucinogens, LSD, PCP, ecstasy), pain medications, tranquilizers, stimulants, or sedatives when use was not directed by a doctor, or tobacco (cigarettes, cigars, pipes, or smokeless tobacco).

“Cannabis use with other substances” was defined as reported past 30-day use of cannabis, *plus* reported use of at least one of the following substances in the past 30 days: alcohol, illicit drugs (including cocaine, crack, heroin, hallucinogens, LSD, PCP, ecstasy), medications when use was not directed by a doctor (pain medications, tranquilizers, stimulants, or sedatives), or tobacco (defined as use of cigarettes, cigars, pipes, or smokeless tobacco). Blunt users were included if respondents self-identified as tobacco users or reported use of tobacco-only products.

#### Age

Given that the prevalence of cannabis use is highest among young adults (where initiation patterns may still be occurring), and generally declines with age, we stratified analyses based on the following age groups: 18–25 years, 26–49 years, 50 years, and older (Substance Abuse and Mental Health Services Administration 2020).

#### Other variables

Analyses also included age at first cannabis use (dichotomized to < 16 years of age vs. > = 16 years), frequency of cannabis use in the past 30 days (dichotomized to < 20 of the past 30 days vs. 20 or more days), whether any past year cannabis use was recommended by a doctor or other healthcare professional (categorized into the following: all, some but not all, none), and past year cannabis dependence (assessed by having to have met 3 or more of a list of 6 dependence criteria from the *Diagnostic and Statistical Manual of Mental Disorders* 4th Edition revised (American Psychiatric Association 2000) assessing tolerance, ability to limit use, ability to stop or cut down on use, continued use despite physical or emotional problems, and reduced participation in important activities).

The following sociodemographic variables were included in analyses: sex, race/ethnicity, education, income, and health insurance coverage. Other variables included were: overall health and past year any mental illness. The variable for past year any mental illness, which was defined as having any past year serious, moderate, or mild mental illness, is a calculated variable that is part of the NSDUH database through modeling of NSDUH data that accounts for responses to a modified version of the World Health Organization Disability Assessment Schedule (WHODAS) that contained eight items found to capture the information represented in the full 16-item scale with no loss of information (Ustun et al. 2010; Center for Behavioral Health Statistics and Quality 2019). Scores from the WHODAS were used in a prediction model that is described more fully in the NSDUH methodology report, with cut points identified in relationship to predictions for mild, moderate, and serious mental illness (Center for Behavioral Health Statistics and Quality 2019).

### Analysis

Weighted frequencies and 95% confidence intervals (CI) were computed for all sociodemographic and cannabis-related variables, overall, and across the two categories of cannabis users, stratified by age. Binary and multivariable logistic regression models were constructed to assess overall correlates of past 30-day cannabis-only use compared with use of cannabis and other substances. Analyses were conducted using SAS University Edition. Proc survey commands were used to account for the complex sampling design of NSDUH.

We were unable to explore historical patterns of substance use among cannabis users because the proportion of past 30-day cannabis users without any other lifetime substance use was so small (< 125 people).

The public-use version of the NSDUH dataset includes imputed variables for major demographics. However, the following variables had missing data: age at first use (missing 37 people from denominator), marijuana use frequency in the past 30 days (missing 205 people from denominator), and recommended use by a healthcare professional (missing 95 people from denominator).

## Results

The prevalence of past 30-day cannabis-only use versus cannabis use and other substances varied by age, with 18–25 years old reporting the highest prevalence of cannabis use of any age group, including the highest prevalence of cannabis-only use and the highest prevalence of cannabis and other substance use (2.0% of 18–25 year old reported cannabis-only use, whereas 20.2% reported using cannabis and other substances in the past 30 days; 0.7% of adults ages 26–49 years reported cannabis-only use, whereas 11.5% reported past 30-day cannabis use and other substances; and 0.6% of adults ages 50 years and older reported cannabis-only use, whereas 4.3% reported past 30-days cannabis use and other substances (Table 1)).

**Table 1:** Prevalence of past 30-day cannabis use^a^ among US adults with and without use of other substances^b^, National Survey on Drug Use and Health, 2017–2018

	No cannabis use (last 30 days) ***N*** = 73,437 Wt% (95% ***CI***)	Cannabis use with other substance use ***N*** = 11,163 Wt% (95% ***CI***)	Cannabis-only use ***N*** = 980 Wt% (95% ***CI***)	Row total
18–25	77.8 (77.2, 78.4)	20.2 (19.7, 20.9)	2.0 (1.8, 2.2)	100%
26–49	87.7 (87.3, 88.2)	11.6 (11.1, 11.9)	0.7 (0.6, 0.9)	100%
50 and older	95.1 (94.7, 95.5)	4.3 (3.8, 4.7)	0.6 (0.5, 0.8)	100%
All adults	89.7 (89.4, 90.0)	9.4 (9.1, 9.7)	0.9 (0.8, 1.0)	100%

^a^The National Survey on Drug Use and Health (NSDUH) survey instrument uses the term “marijuana or hashish.” ^b^Other substances included any past 30-day use of the following: any alcohol, tobacco (cigarettes, cigars, pipes, smokeless tobacco); illicit drugs (cocaine, crack, heroin, hallucinogens, LSD, PCP, ecstasy, ketamine, DMT/AMT/Foxy, Salvia, inhalants, methamphetamine); and pain medications, tranquilizers, stimulants, or sedatives (when use was not directed by a doctor)

When adults with past 30-day cannabis use were considered, 8.4% reported using only cannabis during the same period (data not reported in table). Most adults with past 30-day cannabis use reported past 30-day use of both cannabis and other substances (91.6%, *n* = 11, 163).

### Demographic characteristics of cannabis users with and without other substance use, by age

Stratifying data by age, a higher proportion of adults with past 30-day cannabis use were male across all age groups and cannabis-only and cannabis and other substance use groups (Table 2). Across all age groups, a higher proportion of non-Hispanic blacks reported cannabis-only use, compared with use of cannabis and other substances.

**Table 2:** Demographics of adults with past 30-day cannabis^a^ use, with and without use of other substances^b^, stratified by age, National Survey on Drug Use and Health, 2017–2018

	Cannabis use, past 30-day other substance use			Cannabis-only use		
	18–25 years old ***n*** = 5524	26–49 years old ***n*** = 4799	> = 50 years old ***n*** = 840	18–25 years old ***n*** = 536	26–49 years old ***n*** = 325	> = 50 years old ***n*** = 119
	Wt % (95% ***CI***)	Wt % (95% ***CI***)	Wt % (95% ***CI***)	Wt % (95% ***CI***)	Wt % (95% ***CI***)	Wt % (95% ***CI***)
Sex						
Male	55.5 (53.9, 57.1)	60.9 (59.0, 62.9)	63.0 (57.6, 68.3)	58.1 (52.1, 64.0)	61.4 (54.4, 68.5)	67.2 (56.7, 77.7)
Female	44.5 (42.9, 46.0)	39.7 (37.1, 41.0)	37.0 (31.7, 42.4)	41.9 (36.0, 47.9)	38.6 (31.5, 45.6)	32.8 (22.3, 43.3)
Race						
Non-Hispanic (NH) White	57.7 (56.1, 59.3)	63.9 (62.0, 65.9)	75.7 (72.3, 79.2)	43.2 (37.6, 48.8)	60.0 (53.5, 66.4)	73.8 (62.5, 85.2)
NH Black	14.9 (13.8, 16.1)	15.6 (14.0, 17.2)	12.5 (9.6, 15.5)	24.4 (19.6, 29.3)	15.7 (9.5, 21.9)	13.1 (5.6, 20.6)
NH other	7.5 (6.7, 8.4)	7.3 (6.4, 8.3)	5.7 (4.2, 7.1)	9.6 (6.4, 12.7)	6.9 (3.3, 10.5)	2.7 (0.1, 5.6)
Hispanic	19.8 (18.3, 21.3)	13.2 (12.0, 14.4)	6.1 (4.1, 8.1)	22.8 (18.0, 27.6)	17.4 (11.6, 23.3)	10.3 (0.5, 20.0)
Education						
< high school	12.4 (11.0, 13.8)	11.4 (10.3, 12.4)	13.1 (10.3, 15.8)	20.9 (16.8, 24.9)	13.4 (7.2, 19.7)	8.0 (0.1, 17.6)
High school graduate	29.0 (27.5, 30.5)	23.5 (21.9, 25.2)	28.1 (24.2, 32.1)	37.3 (32.1, 42.5)	26.1 (19.5, 32.8)	29.8 (19.1, 40.5)
Some college/associate degree	44.7 (42.9, 46.5)	34.8 (33.4, 36.3)	30.0 (25.5, 34.6)	36.2 (31.2, 41.2)	38.6 (30.8, 46.4)	40.2 (27.6, 52.8)
College graduate	13.9 (12.4, 15.5)	30.3 (28.1, 32.4)	28.8 (24.2, 33.3)	5.7 (3.0, 8.3)	21.9 (15.2, 28.5)	22.0 (11.9, 32.0)
Age at first cannabis use^c^						
< 16 years	43.4 (41.7, 45.1)	46.1 (44.1, 48.0)	42.6 (37.4, 47.9)	34.0 (29.0, 39.0)	45.8 (39.6, 52.0)	34.9 (23.9, 45.9)
≥ 16 years	56.6 (54.9, 58.3)	53.9 (52.0, 55.9)	57.4 (52.1, 62.6)	66.0 (61.0, 71.0)	54.2 (47.9, 60.4)	65.1 (54.1, 76.1)
Cannabis use frequency^c^ (past 30 days)						
< 20 days	55.3 (53.8, 56.8)	54.6 (52.2, 56.9)	42.6 (37.4, 47.9)	70.5 (65.0, 76.0)	54.6 (52.2, 56.9)	34.9 (23.9, 45.9)
20 or more days	44.7 (43.2, 46.2)	45.4 (43.1, 47.8)	57.4 (52.1, 62.6)	29.5 (24.0, 35.0)	45.4 (43.1, 47.8)	65.1 (54.1, 76.1)
Any past year cannabis use recommended by a healthcare professional?^c^						
All	5.0 (4.4, 5.6)	9.9 (8.8, 11.1)	12.9 (10.0, 15.8)	8.3 (5.3, 11.3)	23.4 (17.8, 28.9)	20.1 (11.5, 28.8)
Some, but not all	5.6 (4.2, 6.2)	6.3 (5.2, 7.5)	5.6 (3.2, 8.0)	5.9 (3.0, 8.8)	9.1 (4.3, 13.9)	7.0 (1.1, 12.9)
None	89.8 (88.6, 91.0)	83.7 (82.4, 85.1)	81.5 (77.6, 85.4)	85.8 (81.9, 89.6)	67.6 (60.1, 75.1)	72.9 (63.0, 82.7)
Past year cannabis dependence	16.7 (15.3, 18.2)	7.5 (6.4, 8.5)	3.3 (1.6, 4.9)	9.9 (6.7, 13.1)	8.9 (4.4, 13.3)	0.2 (0.1, 0.5)
Self-reported overall health						
Good to excellent	90.6 (89.6, 91.6)	85.8 (84.4, 87.1)	76.4 (71.7, 81.0)	92.0 (88.9, 95.1)	79.6 (73.5, 85.7)	71.3 (59.4, 83.3)
Fair/poor	9.4 (8.4, 10.4)	14.2 (12.9, 15.6)	23.6 (18.9, 28.3)	8.0 (4.9, 11.1)	20.4 (14.3, 26.5)	28.7 (16.7, 40.6)
Past year any mental illness	36.7 (34.9, 38.5)	36.7 (34.7, 38.7)	28.4 (24.8, 32.1)	29.1 (25.2, 32.9)	33.3 (27.6, 39.0)	23.6 (11.8, 35.4)

^a^The National Survey on Drug Use and Health (NSDUH) survey instrument uses the term “marijuana or hashish.” ^b^Other substances included any past 30-day use of the following: any alcohol, tobacco (cigarettes, cigars, pipes, smokeless tobacco); illicit drugs (cocaine, crack, heroin, hallucinogens, LSD, PCP, ecstasy, ketamine, DMT/AMT/Foxy, Salvia, inhalants, methamphetamine); and pain medications, tranquilizers, stimulants, or sedatives (when use was not directed by a doctor). ^c^Missing < 1% of data

### Substance use characteristics of cannabis users with and without other substance use, by age

A higher proportion of 18–25 years old with cannabis-only use had initiated cannabis use at or after the age of 16 years, compared with 18–25 years old who reported past 30-day cannabis and other substance use (66.0%, vs. 56.6%, Table 2). Compared with other age groups reporting cannabis-only use, those who were 18–25 years reported the highest prevalence of using cannabis < 20 of the past 30 days (70.5%, vs. 54.6 of 26–49 year old and 34.9% of those > = 50 years), and those who were > = 50 years old reported the highest prevalence of daily or near daily cannabis use (65.1% reported daily or near daily use, compared with 29.5% of 18–25 years old and 45.4% of 26–49 years old). Compared with those ages 26 and older with past 30-day cannabis-only use, a lower proportion of those with cannabis and other substance use of all ages reported that all of their cannabis use was recommended by a doctor. The prevalence of past year dependence was highest among those ages 18–25 years old with past 30-day cannabis and other substance use, with 16.7% meeting criteria for dependence. The prevalence of dependence was lowest among those with past 30-day cannabis-only use who were ages 50 and older, with 0.2% meeting criteria for dependence. Across both cannabis-only and cannabis and other substance use groups, self-reported health status generally declined by age. Past year prevalence of any mental illness was generally similar across cannabis users’ groups and by age, though a higher proportion of 18-25 year olds with past 30-day cannabis and other substance use had past year any mental illness compared with 18-25 year olds with cannabis only use (36.7% vs. 29.1%).

### Multivariable logistic regression model assessing correlates of cannabis only use (vs. cannabis and other substance use)

In a multivariable logistic regression model of adults with past 30-day cannabis use, correlates of cannabis-only use (vs. use of cannabis and other substances) included age (with younger ages having a higher odds of cannabis-only use compared with those ages 50 years and older), education (with college graduates having a higher odds of cannabis-only use compared with those with less than a high school education), and age at first cannabis use (with those initiating use before 16 years of age having a higher odds of cannabis only use). Other correlates included frequency of use (with those using cannabis on 20 or more of the past 30 days having higher odds of cannabis only use) and past year cannabis use recommended by a healthcare professional (with those with none of their use recommended by a healthcare professional having a higher odds of reporting cannabis only use) (Table 3). Past year cannabis dependence and past year any mental illness were not significant correlates of past month cannabis-only use (vs. use of cannabis and other substances).

**Table 3:** Multivariable logistic regression assessing correlates of past 30-day cannabis^a^-only use (vs. cannabis and other substance use^b^), National Survey on Drug Use and Health, 2017–2018

	***N*** = 11,842 Adjusted odds ratio (95% ***CI***)	***p***-value
Sex		
Male	Reference	0.35
Female	1.1 (0.9, 1.4)	
Age		
18–25 years	1.4 (1.1, 2.0)	< .001
26–49 years	2.1 (1.5, 2.9)	
> = 50 years	Reference	
Race		
Non-Hispanic (NH) White	Reference	0.30
NH Black	0.8 (0.6, 1.0)	
NH other	0.9 (0.7, 1.3)	
Hispanic	0.8 (0.6, 1.1)	
Education		
< high school	Reference	< .01
High school graduate	1.0 (0.6, 1.5)	
Some college/associate degree	1.1 (0.7, 1.7)	
College graduate	1.8 (1.1, 2.8)	
Age at first cannabis use^c^		
< 16 years	1.4 (1.1, 1.6)	< .001
≥ 16 years	Reference	
Cannabis use frequency^c^ (past 30 days)		
20 days	Reference	< .01
20 or more days	1.3 (1.1, 1.5)	
Any past year cannabis use recommended by a healthcare professional?^c^		
All	Reference	< .0001
Some, but not all	1.4 (0.9, 2.2)	
None	2.3 (1.8, 2.9)	
Past year cannabis dependence		
Yes	1.3 (0.9, 1.9)	0.12
No	Reference	
Self-reported overall health		
Good to excellent	Reference	0.48
Fair/poor	0.9 (0.6, 1.2)	
Past year any mental illness		
Any	1.2 (0.9, 1.5)	0.18
None	Reference	

^a^The National Survey on Drug Use and Health (NSDUH) survey instrument uses the term “marijuana or hashish.” ^b^Other substances included any past 30-day use of the following: any alcohol, tobacco (cigarettes, cigars, pipes, smokeless tobacco); illicit drugs (cocaine, crack, heroin, hallucinogens, LSD, PCP, ecstasy, ketamine, DMT/AMT/Foxy, Salvia, inhalants, methamphetamine); and pain medications, tranquilizers, stimulants, or sedatives (when use was not directed by a doctor). ^c^Missing < 1% of data

## Discussion

This is among the first studies to assess the prevalence of past 30-day cannabis-only use among adults as compared with past 30-day use of cannabis and other substances. The main finding of this study is that most adults who use cannabis in the USA also report past 30-day use of other psychotropic and potentially addictive substances. These data document that a minority (8.4%) of adults with past 30-day cannabis use report past 30-day use of cannabis only. The various studies describing individuals who use cannabis to quit or decrease use of other substances may represent a small fraction of adults who use cannabis, underscoring the importance of interpreting findings from convenience samples or from studies using qualitative methods with context and caution (see examples of these studies in Corroon Jr. et al. 2017; Lucas 2012; Lucas and Walsh 2017; Mikuriya 2004; Reiman et al. 2017; Sexton et al. 2016).

This study identifies a number of demographic and substance use correlates of cannabis-only use. For example, cannabis-only use is more likely among young adults. More research is warranted to assess the phenomenon. Young adults may still be in an initiation phase in terms of their substance use trajectory. For example, these data suggest that a higher proportion of young adults who reported cannabis-only use initiated their use of cannabis at or after age 16 compared to young adults using cannabis and other substances. Existing research supports the initiation phase as a possible explanation. For example, using nationally representative data, Keyes et al. (2019) found that the age of onset of alcohol and tobacco use had increased over the last four decades, while cannabis initiation age had remained stable or decreased, displacing alcohol and tobacco. Though not significant in a multivariable model, bivariate data from this study suggest that a higher proportion of young adults who report cannabis-only use are black, compared with young adults using cannabis and other substances. A study of NSDUH data from 2004 to 2014 by Fairman et al. (2019) found that African-American youth had higher odds of initiating cannabis use before tobacco use, compared with white youth.

Another significant correlate of cannabis-only use was education, with adults who had past 30-day use of cannabis and were college graduates being more likely than those with less than a high school education to use cannabis only (versus using cannabis and other substances). This finding aligns with other data that suggest that college graduates in general have lower substance use, in part, because previous substance use may lower chances of enrolling in higher education and also because illicit drug use during college decreases the likelihood of staying in college and ultimately obtaining a college degree (King et al. 2006; Arria et al. 2013).

Curiously, adults who reported that none of their past year cannabis use was recommended by a healthcare professional were more likely to use cannabis only (versus using cannabis and other substances). One might have expected to find the reverse association and to find that cannabis-only use was synonymous with medical use. With cannabis becoming much more accessible as nonmedical, adult use (e.g., recreational) cannabis policies increase across US states, it may be that people are self-treating and are no longer obtaining (or needing) a medical recommendation. It could also be those individuals using cannabis medically are also self-treating with other substances medically. More research is warranted to understand how medical use of cannabis is associated with cannabis and other substance use.

Using cannabis on 20 or more of the past 30 days was also associated with cannabis-only use (versus use of cannabis and other substances). In the context of the other findings, this warrants more explanation. It could be due to more medical use of cannabis (regardless of whether cannabis use was recommended by a healthcare professional). It could also be the result of deliberate decisions to use cannabis instead of other substances like tobacco or alcohol (Harwick et al. 2020).

Regardless of whether they were using cannabis only or other substances, adults generally indicated similar self-reported health and past year mental illness across the age groups, and these variables were not significant correlates of cannabis-only use. Given data that suggest associations between substances and physical and mental health (National Academy of Sciences 2017), this finding warrants more investigation using study designs other than cross-sectional.

This study has at least three limitations that should be considered when interpreting findings. First, NSDUH does not include questions about e-cigarettes or hookah, which are popular and frequently used by young adults (Gentzke et al. 2020). It is possible that the prevalence of cannabis-only use among this younger age bracket would be lower if e-cigarettes and hookah were assessed on NSDUH. Surveillance systems like Monitoring the Future (Miech et al. 2019) or PATH (Watkins et al. 2018), both of which collect data on e-cigarettes and cannabis, could be used to explore the impact e-cigarettes have in reducing the prevalence of “cannabis-only” use in youth and young adults.

Second, data are cross-sectional in nature and prevent exploration of patterns of use that could shed more light on the trajectory of substance use in certain groups. Third, NSDUH does not collect data on the type of cannabis products (e.g., containing tetrahydrocannabinol [THC], containing cannabidiol [CBD]) or how they are consumed (e.g., mode or method of use), which prevents any interpretation of potential harms, benefits, or reasons for use that may come from knowing, for example, the THC to CBD ratio, the THC potency, or the methods of use. For instance, individuals using cannabis medically may tend to choose products that are high in CBD or have similar THC to CBD ratios (Gruber et al. 2018). Reasons for cannabis use (e.g., experimentation, medical use) are also not ascertained in NSDUH data, limiting interpretation of findings among young adults.

## Conclusion

A majority of adults with past month cannabis use are also consuming other substances (e.g., tobacco, alcohol, other drugs); 9.4% of the general population have past month use of cannabis and other substances, whereas < 1% report cannabis-only use. More research is needed to continue to assess interactions that cannabis use has with other substance use including alcohol use, tobacco use, use of other federally illicit substances, and use of prescription medications. In particular, more research is warranted in light of these data to better understand the impacts cannabis may have on cessation or continued use of other substances. Policy decisions should be guided by the information that the vast majority of cannabis consumed in the US takes place alongside other substance use while acknowledging that some adults report using only cannabis.

## Data Availability

This study used secondary data from a de-identified, publicly available data set available from the Substance Abuse and Mental Health Administration. Data is available in this link: https://www.datafiles.samhsa.gov/study-dataset/nsduh-2002-2018-ds0001-nsduh-2002-2018-ds0001-nid18772).

## Abbreviations

ACASI: Audio computer-assisted self-interview
CAPI: Computer-assisted personal interviewing
CBD: Cannabidiol
CI: Confidence intervals
NSDUH: National Survey on Drug Use and Health
SAMHSA: Substance Abuse and Mental Health Services Administration
THC: Delta-9-tetrahydrocannabinol
WHODAS: World Health Organization Disability Assessment Schedule
